# Sleep Patterns and Homeostatic Mechanisms in Adolescent Mice

**DOI:** 10.3390/brainsci3010318

**Published:** 2013-03-19

**Authors:** Aaron B. Nelson, Ugo Faraguna, Jeffrey T. Zoltan, Giulio Tononi, Chiara Cirelli

**Affiliations:** 1 Department of Psychiatry, University of Wisconsin-Madison, Madison, WI 53719, USA; E-Mails: abnelson2@wisc.edu (A.B.N.); ugofaraguna@gmail.com (U.F.); jeffreyzoltan@gmail.com (J.T.Z.); giulio.tononi@gmail.com (G.T.); 2 Neuroscience Training Program, University of Wisconsin-Madison, Madison, WI 53706, USA

**Keywords:** adolescence, cerebral cortex, sleep deprivation, slow wave activity

## Abstract

Sleep changes were studied in mice (*n* = 59) from early adolescence to adulthood (postnatal days P19–111). REM sleep declined steeply in early adolescence, while total sleep remained constant and NREM sleep increased slightly. Four hours of sleep deprivation starting at light onset were performed from ages P26 through adulthood (>P60). Following this acute sleep deprivation all mice slept longer and with more consolidated sleep bouts, while NREM slow wave activity (SWA) showed high interindividual variability in the younger groups, and increased consistently only after P42. Three parameters together explained up to 67% of the variance in SWA rebound in frontal cortex, including weight-adjusted age and increase in alpha power during sleep deprivation, both of which positively correlated with the SWA response. The third, and strongest predictor was the SWA decline during the light phase in baseline: mice with high peak SWA at light onset, resulting in a large SWA decline, were more likely to show no SWA rebound after sleep deprivation, a result that was also confirmed in parietal cortex. During baseline, however, SWA showed the same homeostatic changes in adolescents and adults, declining in the course of sleep and increasing across periods of spontaneous wake. Thus, we hypothesize that, in young adolescent mice, a ceiling effect and not the immaturity of the cellular mechanisms underlying sleep homeostasis may prevent the SWA rebound when wake is extended beyond its physiological duration.

## 1. Introduction

As in humans, adolescence in rodents is a transitional time during which the physical and behavioral traits of adulthood develop [1,2]. In mice, adolescence spans the period from weaning (~P21) to sexual maturity (P50–60), and can be further subdivided into a “periadolescence” period (P37–48) around the onset of puberty (at 5–7 weeks of age in most mouse strains), preceded by early adolescence (from weaning to P36), and followed by late adolescence (young adults, P49–60) [3]. This age classification, and the overall strength of rodent models including mice for the purpose of comparison or extrapolation to human development, have been validated extensively [1,2,3,4,5]. 

At time of weaning, most of the growth in the rodent cerebral cortex has been completed. For instance, in the rat mature cortical lamination is reached by P8 [6], myelination starts and most blood vessels grow rapidly and become patent after P10, when explosive synaptic growth occurs [7,8], and by P20 extracellular space has decreased significantly [9,10]. Consistent with major maturational changes occurring during the second week, the total power in the EEG signal in all behavioral states increases after P9 [11], and cortical activity increases sharply after P11 [12]. By the time adolescence starts, at the end of the third week, the EEG signals during NREM sleep, REM sleep and wake are similar to from those in adults [11,13].

While structural and electrophysiological aspects of cortical maturation are reasonably well understood, a complete quantitative description of sleep patterns during the entire adolescence period is missing in rodents. Previous studies recorded sleep at a few select times during adolescence, and in some cases only during part of the light phase [11,13,14,15,16,17,18]. Even less characterized is how sleep homeostatic mechanisms develop and change during adolescence. Four studies have been conducted in rats sleep deprived for 2–6 h during adolescence [17,19,20,21]. Consistently, these experiments found evidence of sleep homeostasis well before weaning (as early as P12), as indexed by changes in sleep amount (e.g., increased duration of NREM sleep) and in sleep consolidation (e.g., decreased number of brief arousals) [17,19]. The same studies, however, found that only some time between the third and the fourth week of age NREM slow wave activity, the EEG power in the 0.5–4.5 Hz range during NREM sleep, becomes a reliable marker of sleep homeostasis as described in adults, increasing with time spent awake and declining with time spent asleep. The reasons for this delay remain unclear. Here we performed in mice a comprehensive study of the changes in sleep quantity and quality, as well as in the response to sleep deprivation, from early adolescence to adulthood.

## 2. Results

### 2.1. Sleep in Baseline

#### 2.1.1. Sleep-Wake Patterns during Baseline

**Figure 1:**
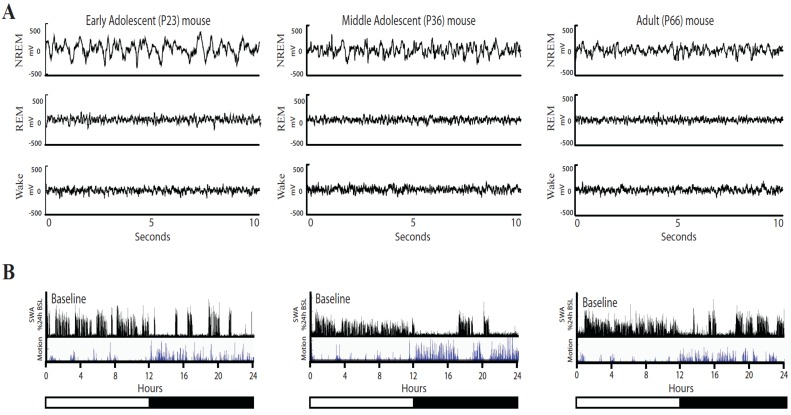
Representative examples of EEG traces in adolescent and adult mice. (**A**) EEG traces (10 s) for NREM sleep, REM sleep, and wake in an early adolescent (P23), middle adolescent (P36) and adult (P66) mouse are shown. (**B**) The bottom panels show the time course of NREM slow wave activity (SWA, expressed as % of 24-h mean SWA, 4-s epochs) and locomotor activity (video-based motion detection, 4-s epochs) for the same animals during 24 h of baseline. Motion is measured in arbitrary units and values cannot be compared across animals.

Fifty-nine male C57/BL-6, yellow fluorescent protein (YFP)-H expressing mice, ages ranging from P15 to P87, were implanted and continuously recorded for 1–3 weeks. Raw EEG traces looked similar in adolescent and adult mice across all behavioral states, except for the greater amplitude of the EEG signals in the younger mice (Figure 1). 

**Figure 2:**
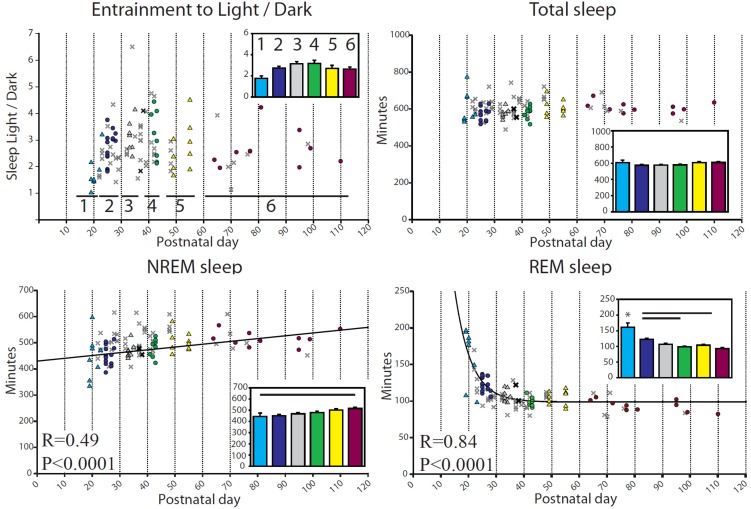
Daily sleep and wake amounts (min/24 h) during baseline for each mouse. In all figures, symbols show values for each mouse, with P and R corresponding to the regression line computed from all animals except those denoted by a faint “X”. Insets depict group means ± SEM, with bars indicating significant differences between groups, and asterisks indicating one group different from all others (*p* < 0.05, Tukey’s HSD). Age groups in all figures: 1≤P25; 2= P26–29; 3= P34–36; 4= P41–44; 5= P50–56; 6≥P60. Age ranges correspond to those chosen for the acute sleep deprivation experiment. The bold “**X**” are baseline days, *i.e.*, the days just before the sleep deprivation day, and are included in the regression analysis. The faint X denotes additional scored days from the same mice that did not qualify as “baseline” (*i.e.*, they occurred >1 day before sleep deprivation, or at least 3 days afterwards). The *x* days are included for reference only but were not used for the ANOVA. In this figure, NREM sleep is fit to age with a linear relationship, *f* = y_0_ + a × *x*, (*f* = min NREM, y_0_ = 429.98, a = 1.08, *x* = Postnatal day) while REM sleep is fit to age with an exponential decay, *f* = y_0_ + a × exp(−b × *x*), (*f* = min REM, y_0_ = 98.80, a = 2012.99, b = 0.17, *x* = Postnatal day).

**Figure 3:**
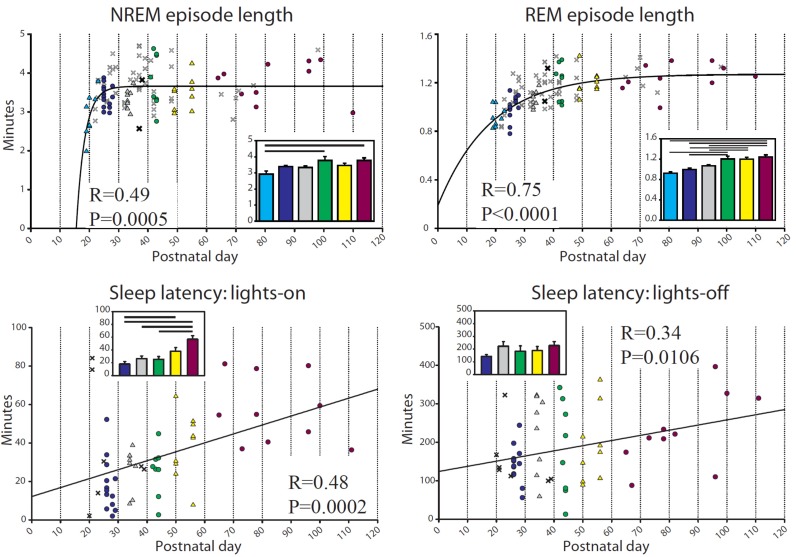
Episode duration and sleep latency during baseline. For episode length, *p* and *R* are from the regression for an exponential equation increasing to a maximum, *f* = y_0_ + a × [1 − exp(−b × x)] (top left: *f* = min, y_0_ = −407.10, a = 410.67, b = 0.32, *x* = Postnatal day), (top right: *f* = min, y_0_ = 0.19, a = 1.08, b = 0.054, *x* = Postnatal day); for sleep latency, *p* and *R* are from the linear regression (*f* = y_0_ + a × *x*) (bottom left: *f* = min, y_0_ = 12.12, a = 0.47, *x* = Postnatal day; bottom right: *f* = min, y_0_ = 120.45, a = 1.41, *x* = Postnatal day).

In the following figures, all sleep and wake parameters are shown for each individual mouse to show interindividual differences, as well as averaged across age groups spanning 3–7 days (except the adult cluster) and “centered” around the day of the sleep deprivation experiment (P < 25 *n* = 8, P26–29 *n* = 14, P34–36 *n* = 8, P41–44 *n* = 8, P50–56 *n* = 9, P > 60 *n* = 10, no group *n* = 2). The age groups were chosen to span the entire adolescent period as comprehensively as possible, but with an emphasis placed on early and middle (peri)adolescence until around puberty (P26–44). All mice older than P25 underwent an acute 4-h sleep deprivation by exposure to novel objects beginning at lights-on, and then were left undisturbed for the next 20 h. The day of the sleep deprivation and the preceding baseline day were used for the statistical analysis to characterize maturational changes in sleep homeostasis. Additional days (*n* = 47) that did not qualify as “baseline” (*i.e.*, they occurred >1 day before sleep deprivation, or at least 3 days afterwards) were scored for a subset of mice. These days are shown in some of the figures (indicated by X) for reference, but were not included in the statistical analysis. In total, sleep staging was performed for 106 baseline days spanning the ages from P19 to P111. To avoid an unnecessarily stressful extended deprivation, mice under P25 underwent a shorter (2 h) sleep deprivation. Individual data points from these mice are shown in the figures but were not included in the ANOVA groups or in the regression analysis; this is why there are six groups for ANOVAs concerning changes in baseline sleep across adolescence, but only five groups for the analysis of the response to 4 h of sleep deprivation. The EEG signal became unusable following the sleep deprivation for three mice under P25 and 1 mouse in the P26–29 age group. Only baseline sleep data for these mice are included (Figure 2, Figure 3 top). Due to poor quality of the signal, only eight mice contributed data from the frontal derivation (P < 25 *n* = 2, P26–29 *n* = 1, P34–36 *n* = 2, P41–44 *n* = 1, P > 60 *n* = 1), and one mouse (P50–56) is included with data only from the parietal derivation.

Mice were entrained to the light/dark cycle early in adolescence, with all age groups showing an average ~2:1 ratio of sleep during the light period relative to the dark period (Figure 2, top left). There was, however, a significant interindividual variability in this ratio, from 1:1 up to 6:1. The total amount of sleep during 24 h did not change from early adolescence to adulthood (599.9 ± 4.76 min, mean ± SEM), while NREM sleep increased slightly across adolescence, showing a weak linear relationship with age that resulted in the youngest mice having significantly less NREM than the adults (Figure 2). Meanwhile, REM sleep decreased steeply during early adolescence, a pattern that was fit well by an exponential decay function (*p* < 0.0001, Figure 2). The amount of REM sleep differed across groups, with the P < 25 mice having significantly more REM sleep than all other groups, and the P26–29 mice having more REM sleep than the adult and P41–44 groups (Figure 2).

The length of NREM and REM sleep episodes increased steeply in the youngest animals before leveling off, a pattern fit by an exponential function increasing to a maximum (Figure 3). Group comparisons showed that the adult level of NREM episode length was reached quickly, with only the <P25 group having shorter bouts than the adults, while adult-like levels for REM episode length were first reached in the P41–44 group (Figure 3). Sleep latency, defined as the time in minutes between lights-on or lights-off and the first episode of consolidated sleep (>1 min), showed a progressive increase with age (Figure 3).

#### 2.1.2. Changes in EEG Power Spectrum during Baseline

**Figure 4:**
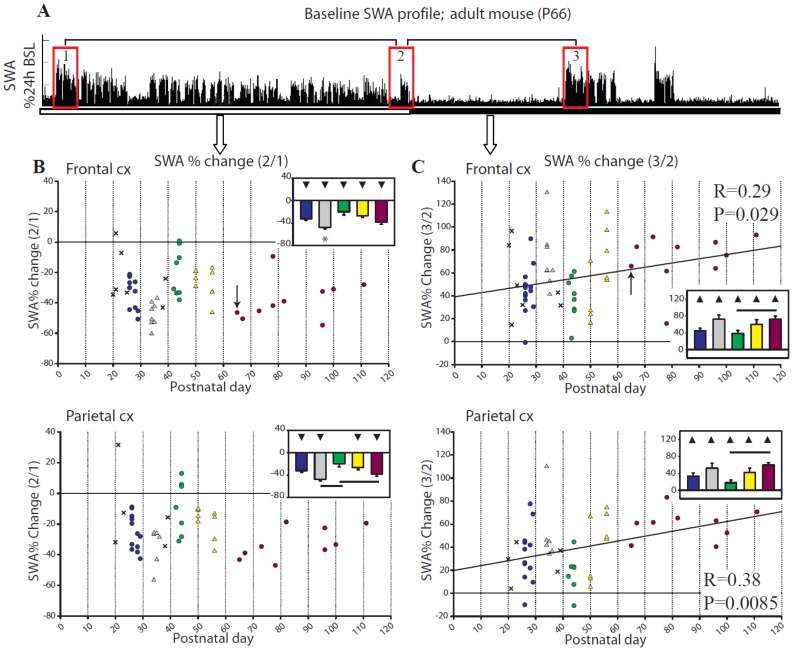
Baseline changes in relative NREM SWA. (**A**) shows changes in relative SWA for the frontal derivation in a representative adult (P66) mouse (indicated by an arrow in **B**,**C**). Boxes 1,2,3 indicate first and last hour of consolidated sleep during the light period, and first hour of consolidated sleep at night, respectively. The ratios 2/1 and 3/2 were used for the analysis shown in (**B**) and (**C**). Bars show differences across groups (Tukey’s HSD), and triangles indicate group means different from 0 (*t*-test; filled symbols *p*<0.05).

In adult mice NREM SWA is a reliable marker of sleep homeostasis: it declines during sleep and increases with time spent awake, although “intensity” of wake and genotype also affect SWA (e.g., [22,23,24]). To determine whether the SWA time course described in adults is also present during adolescence we first quantified the baseline decrease in SWA during the day, the major sleep phase in mice. Specifically, we measured the ratio of NREM SWA in the last hour relative to the first hour of the light period (ratio 2/1 in Figure 4A). In the frontal derivation all groups showed a similar and significant SWA decline during the day (ratio 2/1), with P34–36 mice having a significantly larger decrease than all other groups (Figure 4B, top). Similar changes were present in the parietal derivation, where all groups had a significant decrease except for the P41–44 group, which had a smaller decline than the adult and P34–36 groups (Figure 4B, bottom).

To further assess SWA changes during baseline we calculated the increase in SWA across the extended spontaneous wake period that typically occurs at the light/dark transition; to do so we measured the ratio of NREM SWA in the first hour of consolidated sleep after lights-off relative to the last hour of the light period (ratio 3/2 in Figure 4A). Both in frontal and parietal cortex all groups showed a significant SWA increase, although the P41–44 group had a blunted increase compared to adults (Figure 4C). As was the case for sleep latency, the change in SWA increased linearly with age in both frontal and parietal derivations (Figure 4C).

### 2.2. Sleep after Sleep Deprivation

#### 2.2.1. Changes in Sleep duration after Sleep Deprivation

**Figure 5:**
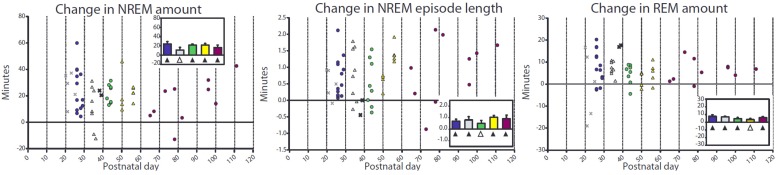
Changes in sleep duration after sleep deprivation. Each faint “X” denotes a mouse that underwent a 2 h deprivation and is included only as a reference. Left and middle panels show the difference (in min) in NREM amount and NREM episode length during the first 4 h of recovery sleep (starting at noon) relative to the first 4 h of baseline sleep (starting at 8 a.m.), while the right panel shows changes in REM amount during the first 8 h of recovery sleep (from noon until lights-off at 8 p.m.) relative to the first 8 h of baseline sleep. Triangles indicate group mean different from 0 (paired 2-tailed *t*-test; filled symbols *p* < 0.05, empty symbols *p* < 0.1).

To further assess SWA changes across adolescence all mice older than P25 were deprived of sleep by exposing them to novel objects for 4 h. During sleep deprivation, which started at light onset, time spent in NREM sleep was in all cases less than 4%, although there was a difference across groups, with the P26−29 group sleeping more than the adults (1.5% ± 0.16% *vs.* 0.2% ± 0.16%). REM sleep never occurred during sleep deprivation. In the first 4 h following sleep deprivation (noon-4pm), NREM sleep amount was significantly increased compared to baseline in all groups except in the P33-36 mice, which only showed a trend (*p* = 0.09); there was no difference across groups (*p* = 0.30, Figure 5, left). Similarly, NREM sleep bout duration increased following sleep deprivation except in the P41–44 mice, which only showed a trend (*p* = 0.054); there was no difference across groups (*p* = 0.68, Figure 5, middle). By the end of the night after sleep deprivation all groups showed a significant NREM deficit (*i.e.*, not all NREM sleep lost during deprivation was recovered), with no difference among them (*p* = 0.42, Table 1). During the light phase (noon–8 p.m.), REM sleep was increased relative to the corresponding time period at baseline in all groups except in the P50–56 mice, which only showed a trend (*p* = 0.052); again there was no difference across groups (*p* = 0.42, Figure 5, right). As was the case for NREM sleep, there was a REM sleep deficit by the end of the dark period following sleep deprivation, with no difference among groups (*p* = 0.24, Table 1). Several other sleep parameters, including number of brief awakenings and duration of REM sleep bouts showed either no changes or small changes in the same direction in all groups (Table 1). In summary, the homeostatic response to sleep deprivation, as measured by increases in sleep amount and sleep bout duration, was present in all groups, with no differences between adolescent and adult mice.

**Table 1:** Changes in sleep parameters following 4 h of sleep deprivation (8 a.m.–12 p.m.). Mean values and standard error (in parenthesis) for each age group are displayed. The deficit in NREM or REM sleep is the difference in minutes between the amount of each sleep phase during the recovery phase (the 20 h of recovery that follow 4 h of sleep deprivation), minus the amount during the 24 h baseline. During the first 20 h following sleep deprivation mice never recovered all the sleep that they lost during sleep deprivation, resulting in a negative value (deficit). The change in REM episode length is the difference in REM episode length for each mouse, as above calculated by subtracting the 20 h mean value during recovery minus the 24 h amount during baseline. The change in brief arousals is the difference in the number of short wake periods (<16 s) per minute of sleep during the first 4 h of recovery sleep after sleep deprivation, minus the number during the first 4 h of baseline sleep. Sleep during the deprivation is the total amount of sleep achieved during the 4 h of sleep deprivation. Sleep attempts during the deprivation is the total number of sleep attempts during the 4 h of sleep deprivation.

Age Group	P26–29	P34–36	P41–44	P50–56	Adults
NREM Deficit (min)	−38.13	−44.60	−43.00	−59.36	−66.91
	(11.11)	(13.96)	(15.77)	(10.66)	(11.09)
REM Deficit (min)	−11.68	−8.32	−12.75	−12.58	−8.72
	(3.04)	(2.56)	(3.80)	(3.26)	(3.68)
Change in REM episode length (min)	0.077	0.097	0.056	0.23	0.29
	(0.059)	(0.044)	(0.068)	(0.081)	(0.069)
Change in Brief Arousals	−0.0043	−0.0046	−0.0017	−0.0020	−0.0054
(number per min sleep 1st 4 h)	(0.0014)	(0.0023)	(0.0031)	(0.0021)	(0.0018)
Sleep during deprivation (min)	1.46	0.64	0.70	0.85	0.20
	(0.16)	(0.26)	(0.29)	(0.34)	(0.16)
Sleep attempts (number during deprivation)	21.77	7.13	9.13	6.78	2.00
	(2.33)	(3.78)	(3.34)	(1.76)	(1.57)

#### 2.2.2. Changes in SWA after Sleep Deprivation

**Figure 6:**
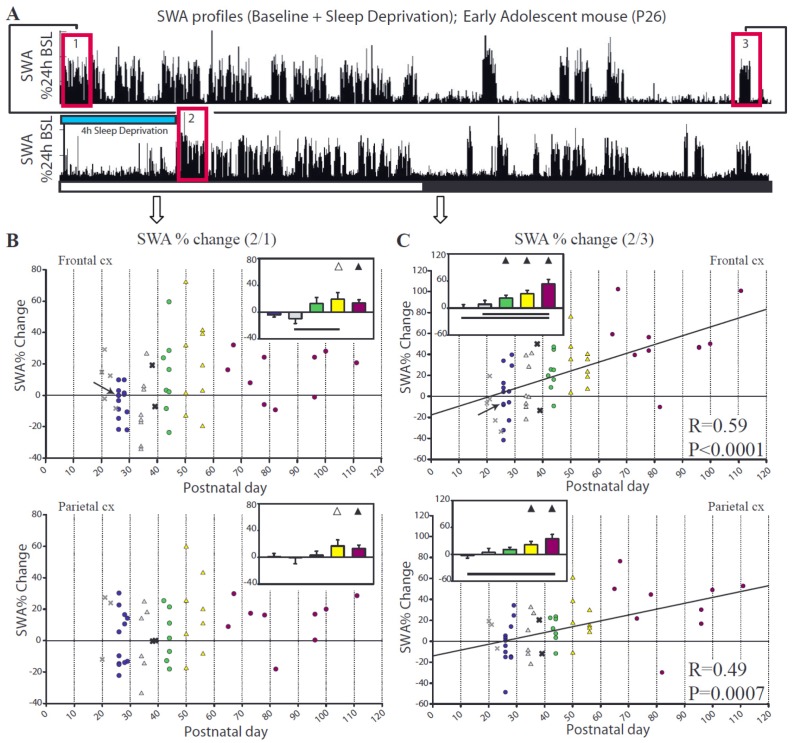
Change in NREM SWA after sleep deprivation. Each faint “X” denotes a mouse that underwent a 2 h deprivation and is included only as a reference. *p* and *R* correspond to the regression line computed from all animals except those denoted by a faint “X”. (**A**) shows changes in relative SWA for the frontal derivation in a representative adolescent (P26) mouse (indicated by an arrow in **B**,**C**). Boxes 1,2,3 indicate the first hour of baseline sleep during the light period, the first hour of recovery sleep after sleep deprivation, and the last hour of baseline sleep at night, respectively. The ratios 2/1 and 2/3 were used for the analysis shown in (**B**,**C**). Bars show differences between groups (Tukey’s HSD), and triangles indicate group means different from 0 (one sample, 2-tailed *t*-test; filled symbols *p* < 0.05, empty symbols *p* < 0.1).

In adults, as expected, SWA in both frontal and parietal cortex increased during the first hour of recovery sleep relative to the first hour of the baseline light period (ratio 2/1, Figure 6A,B). By contrast, the youngest groups of adolescent mice (P26–29, P34–36) showed no group average SWA increase, and several individual animals actually showed a negative SWA rebound (Figure 6A,B). To confirm the lack of a consistent SWA rebound in the youngest mice we also compared SWA levels immediately before and after the period of sleep deprivation (ratio 2/3, Figure 6A,C). Consistent with the previous finding, group average SWA increased after sleep deprivation in adult and older adolescent mice (starting at P41) but not in the younger groups. This change in SWA showed a linear relationship with age for both the frontal (*R* = 0.59, *p* < 0.0001) and parietal (*R* = 0.49, *p* = 0.0007) derivations, such that the older groups (P41-adults) had robust increases in SWA while in the younger mice (P26–29, P34–36) SWA did not significantly increase across at least 4 consecutive hours of wake (Figure 6).

#### 2.2.3. Parameters Affecting SWA Changes after Sleep Deprivation

By examining SWA values for individual animals in Figure 6, it becomes apparent that a large interindividual variability contributes to the lack of a SWA rebound in the younger groups. To understand the source of this variability and tease apart the contribution of multiple parameters, we performed a regression analysis of the percent change in SWA during the first hour after sleep deprivation (Table 2). We first performed this analysis on the frontal derivation, which in adult mice shows the largest SWA rebound after sleep loss [25]. We considered a number of parameters known to affect the SWA rebound, at least in adults, including time spent awake and sleep deprivation efficiency [26,27], REM sleep pressure [28], and changes in the wake spectra (e.g., [29,30,31,32,33,34,35,36,37,38,39]). Because of the inherent variability in maturity levels for individual mice of the same age, we used *weight-adjusted age* (see Experimental Section), which adjusts the age by a factor computed from the weight measured at surgery. Our models focused on the mice spanning adolescence (P26–56, Frontal: *n* = 40 Parietal: *n* = 35). Adult mice exhibited the expected post-deprivation increase in SWA, which did not change as a function of age.

First, we considered the parameters affecting the SWA rebound as classically defined—the increase in SWA during the first hour of recovery sleep relative to the first hour of the baseline light period (ratio 5/1, Table 2, top). The single best predictor of SWA rebound was the ratio between “peak” and “trough” SWA during baseline, i.e. the ratio between SWA during the first hour of the light period, when sleep pressures peaks, and SWA at the end of the major, consolidated sleep period during the light phase (ratio 1/2, Table 2A): animals with a large peak-to-trough ratio were less likely to show a SWA rebound after sleep deprivation. This model was further refined by including the change in the wake alpha EEG power (8–12 Hz) during sleep deprivation, and weight-adjusted age; as both wake alpha and age increased, so did the SWA rebound. Together, these three parameters (decline in baseline SWA, wake alpha, weight-adjusted age), accounted for 43.1% of the variability in SWA rebound. Other parameters, including the efficiency of sleep deprivation and REM sleep amount during recovery sleep, had some value when considered alone but did not significantly improve the model in the presence of stronger parameters. We also tested this model in the two youngest age groups (P26–29, P34–36, *n* = 21), who showed the least SWA rebound. Within this subset of mice the model provided an even better fit, accounting for 56.9% of the variance, and becoming significantly worse if any one of the 3 parameters was removed.

**Table 2:** Multiple predictor linear regression for the SWA rebound after sleep deprivation (**A**,**D**), the SWA changes from immediately before to after sleep deprivation (**B**,**E**), and the SWA changes during spontaneous periods of wake (**C**,**F**). **A**–**C**, frontal cortex; **D**–**F**, parietal cortex. The appropriateness of models to explain the variability in SWA changes was assessed using the Akaike’s information criteria (AIC). For models including multiple predictors, *p*-values indicate the probability that the corresponding parameter has predictive value when all other parameters are included. AIC and *R*^2^ are given for each model; *R*^2^ indicates the portion of variance explained by the model and will increase when additional variables are added to a model. AIC is a measure of the relative goodness of fit for a model. More adequately fitting models have lower AIC, but adding poor predictors will increase AIC (indicating a less appropriate model) even if the *R*^2^ increases.

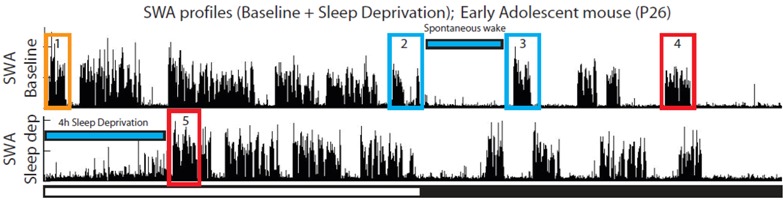			
**(A) Frontal: Predictors for SWA rebound (5/1); % change SWA (5/1) = B_0_ + (predictors)**	***p***	**AIC**	***R*^2^**
Weight-adjusted age (using Gompertz fit)	0.00507	362	0.189
Recovery days from surgery	0.43	370	0.0164
REM/NREM amount (12 h of recovery)	0.0071	363	0.175
*n* of sleep attempts during sleep deprivation	0.053	367	0.0953
Sleep during sleep deprivation (min/4 h)	0.0582	367	0.0913
Brief arousals (*n*/min of sleep, first 4 h of recovery)	0.31	369	0.0273
Increase in wake SWA (1–4 Hz) during sleep deprivation	0.76	370	0.0249
Slow wave energy (SWE = SWA × time) during deprivation (wake and NREM)	0.84	371	0.0011
Time spent awake since last sleep	0.91	371	0.00033
NREM SWA decline in baseline (1/2)	0.000154	355	0.317
NREM SWA decline in baseline (1/2) +	<0.0001	352	0.395
Increase in wake alpha (8–12 Hz)	0.0352		
NREM SWA decline in baseline (1/2) +	0.0009	352	0.400
Weight-adjusted age	0.0297		
NREM SWA decline in baseline (1/2) +	0.00043	352	0.431
Increase in wake alpha (8–12 Hz) +	0.17293		
Weight-adjusted age	0.14348		
**(B) Frontal: Predictors for SWA change from before to after sleep deprivation (5/4); % change SWA (5/4) = B_0_ + (parameters)**	***p***	**AIC**	***R*^2^**
Weight-adjusted age (using Gompertz fit)	0.00025	364	0.30
Recovery days from surgery	0.64	378	0.00595
REM/NREM amount (12 h of recovery)	0.0036	369	0.20
Length of time since last sleep	0.00462	369	0.19
Increase in wake alpha (8–12 Hz)	0.00013	362	0.324
NREM SWA increase in baseline (4/2)	<0.0001	355	0.44
NREM SWA (4/2) +	0.00017	350	0.53
Weight-adjusted age	0.01467		
NREM SWA (4/2) +	0.000037	346	0.575
Increase in wake alpha (8–12 Hz)	0.0015		
NREM SWA (4/2) +	0.00044	344	0.615
Increase in wake alpha (8–12 Hz) +	0.00618		
Weight-adjusted age	0.06158		
NR SWA (4/2) × Increase wake alpha (8–12 Hz) +	0.022	340	0.669
Weight-adjusted age	0.071		
**(C) Frontal: Models Predicting SWA % change (3/2); % change SWA (3/2) = B_0_ + (parameters)**	***p***	**AIC**	***R*^2^**
Weight-adjusted age (using Gompertz fit)	0.913	383	0.0003
Recovery days from surgery	0.39	382	0.0197
Length of time since last sleep	0.016	376	0.14
Increase in wake alpha (8–12 Hz)	0.018	377	0.139
NREM SWA decline in baseline (1/2)	0.000047	365	0.357
NREM SWA decline in baseline (1/2) +	0.00039	365	0.390
Increase in wake alpha	0.1654		
NREM SWA decline in baseline (1/2) +	0.000095	362	0.435
Length of time since last sleep	0.029		
NREM SWA (1/2) × Length of time since last sleep	0.029	358	0.506
**(D) Parietal: Predictors for SWA rebound (5/1); % change SWA (5/1) = B_0_ + (predictors)**	***p***	**AIC**	***R*^2^**
Weight-adjusted age (using Gompertz fit)	0.0045	317	0.219
Recovery days from surgery	0.46	325	0.0165
REM/NREM amount (12 h of recovery)	0.0024	315	0.246
*N* of sleep attempts during sleep deprivation	0.0100	318	0.185
Sleep during sleep deprivation (min/4 h)	0.011	318	0.180
Brief arousals (*N*/min of sleep, first 4 h of recovery)	0.62	325	0.0076
Increase in wake SWA (1–4 Hz) during sleep deprivation	0.581	325	0.0093
Slow wave energy (SWE = SWA × time) during deprivation (wake and NREM)	0.801	325	0.0020
Time spent awake since last sleep	0.967	325	0.0000
NREM SWA decline in baseline (1/2)	0.0015	314	0.266
NREM SWA decline in baseline (1/2) +	0.00048	307	0.446
*N* of sleep attempts during sleep deprivation	0.00292		
NREM SWA decline in baseline (1/2) +	0.012	312	0.360
Weight-adjusted age	0.038		
NREM SWA decline in baseline (1/2) +	0.0033	307	0.470
*N* of sleep attempts during sleep deprivation +	0.0164		
Weight-adjusted age	0.2466		
**(E) Parietal: Predictors for SWA change from before to after sleep deprivation (5/4);** **% change SWA (5/4) = B_0_ + (parameters)**	***p***	**AIC**	***R*^2^**
Weight-adjusted age (using Gompertz fit)	0.0004	319	0.322
Recovery days from surgery	0.51	332	0.0135
REM/NREM amount (12 h of recovery)	0.0004	319	0.320
Length of time since last sleep	0.0046	324	0.219
Increase in wake alpha (8–12 Hz)	0.00059	320	0.304
NREM SWA increase in baseline (4/2)	0.00056	320	0.306
NREM SWA (4/2) +	0.034	316	0.412
Weight-adjusted age	0.023		
NREM SWA (4/2) +	0.0039	313	0.466
Increase in wake alpha (8–12 Hz)	0.0041		
NREM SWA (4/2) +	0.063	311	0.526
Increase in wake alpha (8–12 Hz) +	0.010		
Weight-adjusted age	0.057		
NR SWA (4/2) × Increase wake alpha (8-12 Hz) +	0.100	311	0.567
Weight-adjusted age	0.034		
**(F) Parietal: Models Predicting SWA % change (3/2); % change SWA (3/2) = B_0_ + (parameters)**	***p***	**AIC**	***R*^2^**
Weight-adjusted age (using Gompertz fit)	0.953	334	0.000
Recovery days from surgery	0.47	334	0.016
Length of time since last sleep	0.0060	326	0.207
Increase in wake alpha (8–12 Hz)	0.133	332	0.067
NREM SWA decline in baseline (1/2)	0.0018	324	0.258
NREM SWA decline in baseline (1/2) +	0.0061	326	0.265
Increase in wake alpha	0.5839		
NREM SWA decline in baseline (1/2) +	0.00084	316	0.444
Length of time since last sleep	0.00260		
NREM SWA (1/2) × Length of time since last sleep	0.026	312	0.527

Next, we considered the parameters affecting the increase in SWA from immediately before to after the period of sleep deprivation (ratio 5/4, Table 2, top). The best predictor for this SWA increase, accounting for 44% of the variance, was the ratio between the SWA immediately preceding the onset of sleep deprivation, at the end of the dark period during baseline, and the SWA at the end of the major sleep period during the light phase (ratio 4/2, Table 2B); in other words, in the mice with a large increase in SWA during the baseline dark phase, SWA was less likely to increase from immediately before to after the period of sleep deprivation. As before, other relevant predictors included the increase in wake alpha EEG power across the sleep deprivation and weight-adjusted age. Together, these 3 parameters accounted for 61.5% of the variance, which increased to 66.9% when an interaction was allowed to occur between the increase in wake alpha and the SWA decline in baseline: the larger was the increase in wake alpha, the less negative was the effect of the baseline SWA decline (Table 2B).

Finally, we considered the change in SWA across the consolidated periods of spontaneous wake that typically occur at the light/dark transition during baseline (ratio 3/2, Table 2, top). Again, the single best predictor was the peak-to-trough SWA ratio during the baseline light phase (ratio 1/2) but, in this case, the larger was the decrease in NREM SWA across the light period, the *larger* was the increase across the following wake period (Table 2C). The time spent awake at the light/dark transition (between 2 and 3 in Table 2, top; see also Figure 3 bottom, sleep latency: lights-off) was significantly related to the increase in SWA and accounted for 14% of the variance. The SWA decline during the light phase combined with time spent awake explained 44% of the variance. Weight-adjusted age had no predictive value, and the increase in alpha during spontaneous wake had some value (*R*^2^ = 0.139) as a single predictor. 

Then, we examined whether the same parameters affect SWA in the parietal cortex (Table 2D–F). As was the case for the frontal derivation, the single best predictor of the SWA rebound was the ratio between “peak” and “trough” SWA during baseline (Table 2D). Unlike for the frontal derivation, however, the change in alpha EEG power was not a significant predictor (*p* = 0.44), and the most appropriate parietal model, which explained 47% of the variability in the SWA rebound, included SWA decline, weight-adjusted age, and number of sleep attempts rather than wake alpha power (Table 2D). The significant predictors for the increase in SWA across sleep deprivation (Table 2E), and those for the change in SWA across spontaneous wake (Table 2F), were exactly the same as in the frontal derivation.

**Figure 7:**
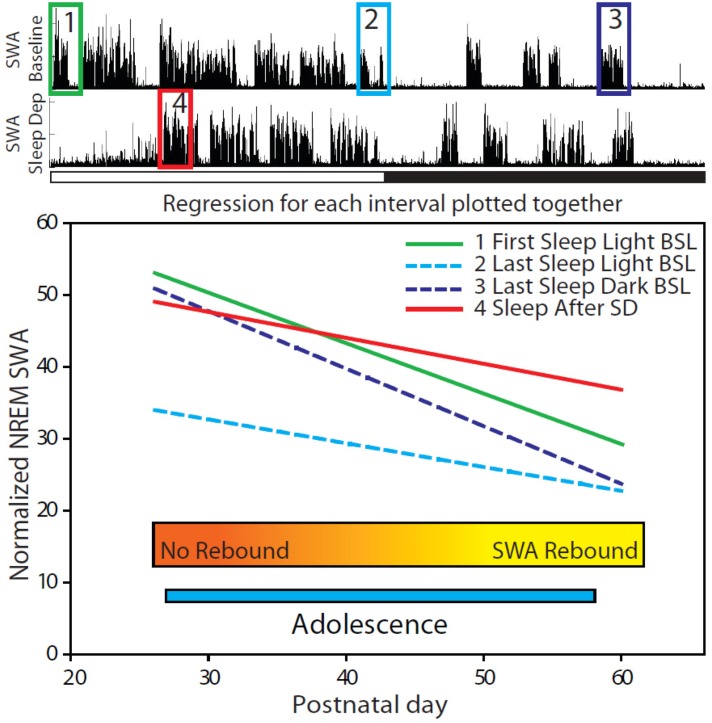
Relationship between age and mean frontal SWA values for 4 daily time points. Plotted is the line of best fit (*f* = y_0_ + a × *x*) (*f* = normalized SWA, y_0_ = intercept, a = slope (SWA/postnatal day), *x =* Postnatal day) for each specified interval (Age *vs.* Normalized NREM SWA) for adolescent mice (P26–56, *n* =40). NREM SWA was normalized for the mean power density in the 15–30 Hz range for each day. All lines represent statistically significantly relationships (*p* < 0.05).

Overall, this analysis revealed that the most important predictor of the change in SWA after sleep deprivation is the extent of SWA changes during baseline. Specifically, whether or not a mouse had an increase in SWA in response to a sleep deprivation was primarily determined by the magnitude of the decline in SWA during the light period, as well as by increase in SWA during the dark period. To understand why, we measured how the difference in SWA amplitude between younger and older mice varied as a function of time of day, and found that such difference varied significantly across the 24-h cycle, as shown in Figure 7. Specifically, the high pressure SWA values, reached at the onset of the light phase, differed much more across age groups than the low pressure SWA values, reached at the end of the major sleep phase. Indeed, considering the lines of best fit for each interval, the expected initial value for NREM SWA on the baseline day decreased from 53.9 for P25 mice to 29.3 for adult mice at P60, which represents a decrease of 0.703 per day across the entire adolescent period. Meanwhile at low sleep pressure, NREM SWA decreased by only 0.332 per day, from 34.3 at P25 to 22.7 in adult mice. Thus, younger mice can reach higher peak SWA values during baseline than adults, but after the sleep pressure is discharged their SWA reaches low levels not very different from those of adults. Of note, Figure 7 also shows that during the last sleep episode of the dark period (box 3), older mice have SWA that is near their trough value (box 2), while younger mice are already near their peak value (box 1). 

#### 2.2.4. Other Changes in the EEG Power Spectrum after Sleep Deprivation

**Figure 8:**
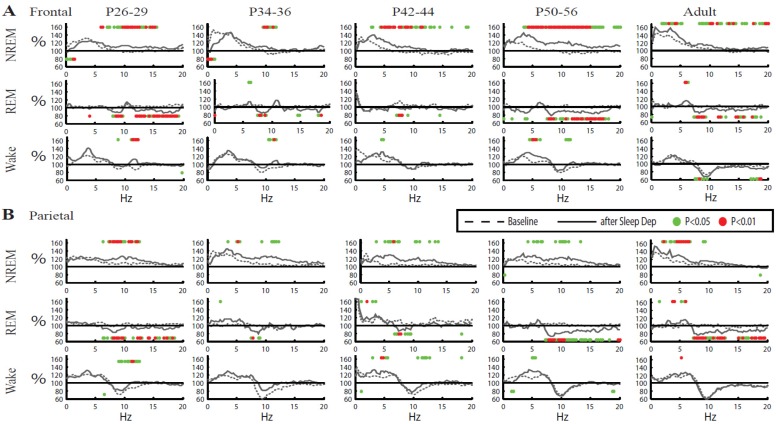
Overall changes in EEG power spectra after sleep deprivation for all vigilance states. Mean EEG spectra for NREM sleep, REM sleep and wake during baseline (dotted line) and sleep deprivation (solid line) are plotted in 0.25 Hz frequency bins. The mean of each frequency bin is expressed relative to its 24-h baseline mean. Spectra for NREM sleep refer to the first hour from sleep onset, when most SWA rebound occurs. Since little REM sleep occurs during the first hour of recovery, spectra for REM sleep and wake are instead mean of the first 4 h from sleep onset. Dots above and below traces indicate significant increases and decreases, respectively, after sleep deprivation *vs.* baseline (*p* < 0.05 red, *p* < 0.01 green; one sample, 2-tailed *t*-test).

To determine whether the differences between adult and adolescent mice described above were specific for NREM SWA we examined the whole spectra (0.1–30 Hz) in all three behavioral states, NREM sleep, REM sleep, and wake (Figure 8). In both frontal and parietal cortex of adults and older adolescent mice the increase in NREM SWA after sleep deprivation was accompanied by significant increases in a broad range of higher frequencies. By contrast, during NREM sleep the youngest mice (P26–29, P34–36) showed either a significant decrease (frontal) or no change (parietal) in the SWA range after sleep deprivation, despite still showing increases in higher EEG frequencies, usually centered around 10 Hz (Figure 8). Neither frontal nor parietal cortex showed changes in the SWA range during wake or REM sleep, in any age group. Instead, at all ages the post-deprivation REM sleep spectra showed a decrease at around 10 Hz, while there was no clear pattern during wake. Overall, these results suggest that the post-deprivation lack of an SWA rebound in some adolescent mice is restricted to the lowest EEG frequencies and specific for NREM sleep.

## 3. Discussion

The main goal of this study was to provide a comprehensive analysis of the changes in sleep pattern in adolescent mice, and to try to clarify previously unanswered questions relative to the development of sleep homeostasis as measured by SWA. We found few changes in sleep pattern across adolescence, the major one being a significant decline in the amount of REM sleep, while the duration of total sleep remained constant and NREM sleep increased slightly. Human studies have generally reported no change in total sleep time across adolescence when subjects were not forced to anticipate wake-up time due to school schedules, and significant declines in total sleep time and NREM sleep time only during schooldays [40,41,42]. This suggests that psychosocial factors are a major determinant of sleep time in adolescents, and may affect NREM sleep especially. The lack of such psychosocial factors in our mice, recorded in isolation in a laboratory cage, may explain why total time spent asleep remained constant in our study from early adolescence to adulthood, and NREM sleep showed a small trend to increase with age. Several human studies have also reported a decrease in the deepest stages of NREM sleep (slow wave sleep, stages 3 and 4, N3) and an increase in stage 2 (N2) (e.g., [43,44,45,46]). Since NREM sleep in rodents is not traditionally subdivided in substages, we do not know whether a similar switch occurred in our mice throughout adolescence. REM sleep, on the other hand, showed no change throughout adolescence in some reports [43,44,47], and an increase during both school days and weekends in a recent longitudinal study [42]. In our experimental conditions, in which mice could fall asleep whenever they wanted, there was a clear developmental decline in REM duration. This decline, however, was not gradual, but happened quickly within a few days during early adolescence, well before the onset of puberty (at ~P40), and afterwards REM sleep amounts were stable. This early steep decline in REM sleep may be similar to the decrease in REM sleep occurring in children before 8 years of age [47]. Of note, in our mice the steep decline in REM sleep occurred while total time spent asleep remained constant, suggesting that even as there was a relative shift from REM to NREM sleep need, the overall sleep requirement remained relatively constant across early adolescence. We also found that the duration of NREM sleep episodes showed a steep and early increase, reaching adult-like levels already during early adolescence, while REM episode length showed a slower, more progressive increase (Figure 3). Feinberg [48] observed that the duration of slow wave sleep episodes in humans decreases from 7–13 years to 21 years of age and then remains stable, while the duration of REM sleep episodes does not change from childhood to adulthood. It is difficult, however, to directly compare the human data with ours, because they are strictly dependent on the minimum duration criteria used to define a single episode; moreover, in humans only slow wave sleep was considered, rather than the entire NREM sleep episode as in our case.

Previous studies found that throughout adolescence, and in some cases even well before weaning [49], rats respond to short periods of sleep deprivation by increasing the duration of sleep and decreasing sleep fragmentation [17,19,20,21]. The same studies, however, found that NREM SWA shows different, if not opposite, temporal profiles before and after P24 [17,19,20]. Specifically, one study found that SWA already shows an adult-like profile at P24, increasing during the major wake period (dark phase) and declining during the major sleep period (light phase), while it progressively increases during both the light and dark phase at P12 and P16, and at P20 the 24-h SWA time course is almost flat [13]. Another study found rapid, ultradian declines in SWA across a few hours of sleep at P23, but no progressive decrease during the light phase, and very high SWA levels at night [17]. Moreover, at P24 SWA was found to strongly increase after sleep deprivation, to an extent even larger than at P30 [17], while the SWA rebound was absent at P12 and P16 and small at P20 [19,20]. Finally, more recent studies found evidence for a homeostatic SWA response after sleep deprivation at both P22 and P30 [21] and, in response to mild chronic sleep restriction (4 h/day), in both adult (P65–72) and adolescent (P29–34) rats [50]. Thus, it seems that, in rats, it is only some time between the third and the fourth week of age that SWA reliably increases across extended periods of wake. Why this does not occur before P24 is unclear, since, as mentioned above, most maturational changes in rodent cortex are completed by the end of the second week of age. It has been proposed that developmental changes in brain metabolism and adenosinergic signaling could underlie the differential response to sleep deprivation before and after P24, while early cortical synaptogenesis could account for some of the progressive SWA increase at P12 and P16 [13,19]. The studies in rats discussed above reported only average group data. Our experiments in mice show that there is high interindividual variability in the response to sleep deprivation as measured by both frontal and parietal SWA, and identified three parameters that together could explain from 43% to 67% of the variance in the SWA response in the frontal derivation, depending on how the SWA change was calculated: the ratio between “peak” and “trough” SWA during baseline, the increase in the wake alpha EEG power (8–12 Hz) during sleep deprivation, and the weight-adjusted age. While the first parameter was negatively correlated with the SWA rebound, the last two were positively correlated with the SWA response. Thus, the SWA rebound in frontal cortex is more likely in older mice, with a small decline in frontal SWA during baseline sleep, and a large increase in alpha activity during sleep deprivation. The SWA changes measured in the parietal derivation were affected by almost all the same parameters described for the frontal cortex, the only exception being that the number of sleep attempts during deprivation, rather than the wake alpha power, was a significant predictor in parietal cortex for the SWA rebound as classically defined (the first hour of recovery sleep relative to the first hour of the baseline light period). Wake alpha power, however, continued to be a significant predictor for the other two SWA changes that we examined, across sleep deprivation and across spontaneous wake. Previous work in humans found that increased alpha activity in the wake EEG with eyes open may be associated with higher subjective sleepiness and reduced alertness [51], and a more recent study showed that an adenosine deaminase polymorphism that increases slow wave sleep also results in reduced attention and higher wake EEG alpha activity during sleep deprivation [52]. Thus, wake alpha power and number of sleep attempts during deprivation may reflect the same phenomenon, an increase in sleep pressure.

The single best predictor of SWA rebound in adolescent mice was the decline in frontal SWA during the major sleep phase at baseline. To understand why, it is important to remember that our youngest mice have the highest absolute values of SWA (Figure 1), consistent with many studies in human adolescents [53,54,55,56]. Moreover, as summarized in Figure 7, we found that the SWA values at the peak of sleep pressure (at the onset of the light phase) differed much more across age groups than the SWA values after sleep pressure was discharged at the end of the light phase, or at the end of the dark period. Overall, these findings suggest that a ceiling effect may explain, at least in part, the lack of SWA rebound after sleep loss in many adolescent mice. This working hypothesis, however, will need to be tested by other experimental studies.

The few studies in humans that assessed the response to sleep deprivation during adolescence found that relative to older adolescents, prepubertal children show (1) higher absolute values of SWA or other related parameters, such as the slope of slow waves, during baseline, and (2) similar absolute increases but smaller relative increases of these parameters after sleep deprivation [57,58]. For instance, despite similar absolute changes, the average relative increase in NREM SWA after 36 h of sleep deprivation was 39% of mean all-night SWA in Tanner 5 adolescents, compared to 18% in Tanner 1 and 2 adolescents [57]. Similarly, the mean relative increase in the slow wave slope after sleep deprivation was 131% in mature adolescents, relative to 117% in prepubertal children [58]. Of note, Kurth and colleagues [58] show slope values for the first 5 sleep cycles in their Figure 3, from which it is apparent that the difference between younger and older subjects is most obvious in the peak values of the slope reached during the first sleep cycle, and much less prominent, although still significant, in the low pressure values reached at the end of the night. The smaller relative changes in SWA and slope in younger subjects, combined with faster SWA dynamics during wake as estimated by simulations, have prompted the suggestion that children live under higher sleep pressure and closer to “saturation” [57], a conclusion consistent with our findings in mice. Older subjects, on the other hand, could have more headroom for SWA to grow, and thus may afford longer sustained wake and bigger relative SWA increases before reaching saturation.

To understand the mechanisms underlying SWA changes, and how they are affected by age, it is important to distinguish between fast, daily changes in SWA, those that have been classically linked to sleep homeostasis, and slower, developmental changes. In adult mice of the same strain used in this study, we found that daily changes in SWA are not reflected in changes in the number of cortical synapses, although the analysis so far has been limited to the apical dendrites of layer V neurons in one cortical area [59]. In adolescent mice, the same layer V neurons show a net decrease in synapse number after several hours of sleep, and a net increase after several hours of wake [59,60]. Even in adolescents, however, it is likely that most of the daily changes in SWA are driven by changes in synaptic efficacy, rather than synaptic number. Indeed, converging evidence from experiments in both animals and humans show that, at least in adults, high SWA values at sleep onset reflect rapid increases in synaptic efficacy occurring in a matter of few hours due to experience and learning during wake, and that the progressive decline in SWA during sleep reflects a process of synaptic down regulation in many synapses [61,62,63]. Thus, one could speculate that the large absolute difference in SWA that distinguishes younger from older mice during early, high pressure sleep is driven mainly by difference in synaptic efficacy, while the much smaller difference that remains after sleep pressure has been discharged may be due to differences in synaptic density. Testing this hypothesis requires a systematic analysis of changes in SWA, synaptic efficacy, and synapse number across adolescence, and is the subject of a future study.

Despite the lack of SWA increase, the youngest groups of mice (P26–29, P34–36) showed an increase of frequencies around 10 Hz after sleep deprivation, as did all the other experimental groups. In adults, it is well established that the increase in EEG frequencies after sleep deprivation is not confined to the SWA range, but often extends to the theta and alpha band (~0.5–11 Hz; e.g., [64,65,66]). Classically, theta (5–9 Hz) activity has been described as the wake EEG marker of sleep need [30,32,36], but recent studies show that local changes in wake theta activity correlate with local changes in sleep SWA, and suggest that the same cellular mechanisms may underlie both slow waves and theta waves [67,68]. In human infants (2–9 months old), low range (<1.75 Hz) SWA does not decline monotonically during the night, while theta activity (6.5–9 Hz) does, prompting the suggestion that during early development theta activity during sleep may be able to reflect sleep homeostasis before SWA [69]. Our data are consistent with this interpretation, but also suggest that SWA fails as a homeostatic marker because of saturation. 

## 4. Experimental Section

### 4.1. Recordings of Sleep and Locomotor Activity and Sleep Deprivation

Male YFP-H mice (Jackson Laboratory, Bar Harbor, Maine) were maintained in a colony room on a 12 h light/12 h dark cycle (lights on at 8 a.m.) with food and water available *ad libitum*. Mice (P15–P87) were implanted for chronic polysomnographic recordings under isoflurane anesthesia (1%–2% in 100% O_2_). Prior to surgery, all electrodes were directly soldered to flexible wires (#NUF30-4046, Cooner Wire, Chatsworth, CA, USA). Gold plated miniature screw electrodes (0.7 mm diameter) were placed over the right and left frontal (anteroposterior, AP, +1 mm from bregma; mediolateral, ML, 1 mm), and parietal (AP −2 mm; ML 2 mm) cortices and one over cerebellum (AP −1 mm from lambda) as reference. During placement special care was made to advance the screws the minimum amount to remain fixed (typically <1 turn). Two vinyl-coated braided stainless steel wire electrodes (#AS636, Cooner Wire) were placed in the nuchal muscle for electromyogram (EMG) recording. Electrodes were insulated and affixed to the skull using dental cement. Following surgery, mice were housed individually in sound-attenuating, environmentally controlled recording chambers (12:12 LD, lights on at 8 a.m., 25 ± 1 °C, food and water ad libitum). All electrodes were gathered into a flexible cable and connected to the Multichannel Neurophysiology Recording system (Tucker-Davis Technologies, TDT, Alachua, FL, USA). EEG and EMG signals were collected continuously at a sampling rate of 256 Hz (digitally filtered between 0.1 and 100 Hz). For sleep staging, signals were processed by custom-made Matlab scripts (Mathworks, Natick, MA) using standard TDT routines and subsequently converted into European Data Format (EDF) with Neurotraces software (Fort Lauderdale, FL, USA). Twenty-four hour polygraphic recordings were scored offline for NREM sleep, REM sleep, and wake by visual inspection of 4-s epochs (SleepSign; Kissei Comtec, Irvine, CA, USA) according to standard criteria. Wake was characterized by low voltage, high frequency EEG pattern and phasic EMG activity. NREM sleep was characterized by the occurrence of high amplitude slow waves and low tonic EMG. During REM sleep the EEG was similar to that during wake, but only heart beats and occasional twitches were evident in the EMG signal. NREM and REM episodes were defined according to criteria that allow brief interruptions (≤16 s) but require that the vigilance state accounts for >80% of the episode length [70,71]. The average EEG power spectra for the fronto-cerebellar and parieto-cerebellar derivations (0.0−30 Hz) was computed using averaged periodograms from an FFT routine using consecutive 4-s Hanning windows. Absolute SWA (0.5−4.0 Hz) and SWA relative to NREM high frequencies (>15 Hz), a previously described normalization [72], were computed. The normalization to high frequencies was used to control for possible daily changes in signal strength due to technical issues (progressive deterioration of the signal, skull growth, *etc*.). Motor activity was quantified by custom-made video-based motion detection algorithms with a time resolution of 1 s (Matlab) [59]. Acute sleep deprivation started at light onset, and lasted 2 (at ages <P25) or 4 h (all other ages). Mice were kept awake for up to 4 h by introducing novel objects into their cages. 

For all mice having surgery before P20 special care was taken to make sure they received proper nutrition, following the recommendation of the veterinary staff. Specifically, in addition to the normal food pellets (given to allow chewing/gnawing), these mice received the breeder diet (8626 Teklad Mouse breeder diet, Harlan laboratories, Madison, WI, USA), which is softer and has a higher fat content. Additionally, 2 pellets of breeder diet were softened in water and replaced daily with fresh softened pellets until there was clear visual evidence of the solid pellets being consumed. Each mouse was given special bedding material made from pulped cotton fiber for maintaining warmth when single housed (NestlestsT, Ancare, Bellmore, NY, USA). For the youngest animals a portion of the bedding from the home cage was transferred to the recording cage so that a nest was available immediately after surgery. 

All animal procedures followed the National Institutes of Health Guide for the Care and Use of Laboratory Animals and facilities were reviewed and approved by the IACUC of the University of Wisconsin-Madison, and were inspected and accredited by AAALAC.

### 4.2. Weight-Adjusted Age

During the early postnatal period in mice recording the age in days is only an approximate indicator of maturation due to a number of difficult to control variables including, health of the mother, litter size and competition for maternal nourishment between siblings [73]. Thus, even if the exact moment of birth is known, age in days becomes only an approximation of actual maturity. In order to remove some of the variability associated with this uncertainty we transformed our approximate measurement of age into a more continuous and accurate indicator of maturity by combining the age at sleep deprivation and the weight recorded at the time of surgery. The surgery weight was satisfactorily fit to a Gompertz curve (*R*^2^ = 0.9420, *p* < 0.0001) giving the expected maturational curve for the mice in this study. We chose to fit surgery weight to a Gompertz curve because it is an asymmetrical sigmoid curve that allows for the higher valued asymptote to be reached more slowly than the lower valued asymptote. This pattern is also clearly beneficial for weight across adolescence since the mice initially grow quickly but then continue to gain weight at a slower rate into adulthood. Additionally, a recent study in humans extensively used the inverse of a Gompertz curve to describe the maturational change in SWA during adolescence [74]. From this expected curve we estimated the actual age at surgery by solving the Gompertz equation for age.

Surgery Weight Age = −9.98 × −ln{ln[(Surgery Weight − 4.12)/20.17]} + 21.68

A weight adjusted age for the day of sleep deprivation was determined by adjusting the sleep deprivation age by the number of days each mouse was ahead or behind the Gompertz growth curve at surgery. This weight-adjusted age was used in the models shown in Table 2 to explain the variability in the % increase SWA, because it was a better predictor than either weight or age alone, and because it simplified the models by condensing two parameters of maturity (weight and age) into a single value. For the sake of clarity only models with weight-adjusted age have been included in Table 2. In the figures and corresponding statistical analyses, instead, age indicates chronological age. 

### 4.3. Statistical Analysis

Regression analysis was performed using R (R Foundation for Statistical Computing, Vienna, Austria); all other statistics were performed using Matlab 2007 statistics toolbox (Mathworks, Natick, MA, USA). Figures depicting changes in sleep parameters *vs.* age were created using SigmaPlot (Systat Software, Inc., San Jose, CA, USA) and were fit to linear models except when exponential functions were superior at reducing the sum of squares and producing an even residual distribution. Baseline sleep parameters and the response to sleep deprivations were analyzed both using age as continuous variable for regression and as a grouping criterion for one-way ANOVA when sleep deprivations occurred. Scored days that did not fit within an age cluster were included in the regression but not the ANOVA. If significance *p* < 0.05 was reached the ANOVA was followed by post-hoc Tukey’s honestly significant difference test. Differences between baseline and sleep deprivation were assessed by paired 2-tailed *t*-tests. Whether the % change in SWA differed from 0 was assessed by one-sample 2-tailed *t*-tests.

## 5. Conclusions

Our results show that adolescent mice respond to sleep deprivation in a way very similar to adults, by sleeping longer and with more consolidated sleep bouts. The ability of adolescent mice to show a significant rebound in SWA after sustained periods of wake is strongly and negatively affected by their high absolute values of SWA at sleep onset. Thus, the absence of a SWA rebound after sleep deprivation most likely reflects a ceiling effect rather than the immaturity of the cellular mechanisms—whether they be of metabolic, hormonal, and/or plastic nature—that underlie sleep homeostasis. This conclusion is supported by the fact that as long as wake duration remains within the physiological limits, as during baseline, SWA shows the same expected homeostatic changes in adolescents and adults, declining in the course of sleep and increasing across periods of spontaneous wake.
